# Machine learning risk prediction model for acute coronary syndrome and death from use of non-steroidal anti-inflammatory drugs in administrative data

**DOI:** 10.1038/s41598-021-97643-3

**Published:** 2021-09-15

**Authors:** Juan Lu, Ling Wang, Mohammed Bennamoun, Isaac Ward, Senjian An, Ferdous Sohel, Benjamin J. W. Chow, Girish Dwivedi, Frank M. Sanfilippo

**Affiliations:** 1 Medical School, The University of Western Australia, Perth, 6009 Australia; 2 School of Population and Global Health, The University of Western Australia, Perth, 6009 Australia; 3 Department of Computer Science and Software Engineering, The University of Western Australia, Perth, 6009 Australia; 4 Harry Perkins Institute of Medical Research, Murdoch, 6150 Australia; 5 The State Key Laboratory of Integrated Service Networks, Xidian University, Xi’an, 710071 China; 6 School of Electrical Engineering, Computing and Mathematical Sciences, Curtin University, Bentley, 6102 Australia; 7 Discipline of Information Technology, Murdoch University, Murdoch, 6150 Australia; 8 Division of Cardiology, University of Ottawa Faculty of Medicine and University of Ottawa Heart Institute, Ottawa, ON K1N 6N5 Canada; 9 Cardiology Department, Fiona Stanley Hospital, Murdoch, 6150 Australia

**Keywords:** Machine learning, Acute coronary syndromes, Drug safety

## Abstract

Our aim was to investigate the usefulness of machine learning approaches on linked administrative health data at the population level in predicting older patients’ one-year risk of acute coronary syndrome and death following the use of non-steroidal anti-inflammatory drugs (NSAIDs). Patients from a Western Australian cardiovascular population who were supplied with NSAIDs between 1 Jan 2003 and 31 Dec 2004 were identified from Pharmaceutical Benefits Scheme data. Comorbidities from linked hospital admissions data and medication history were inputs. Admissions for acute coronary syndrome or death within one year from the first supply date were outputs. Machine learning classification methods were used to build models to predict ACS and death. Model performance was measured by the area under the receiver operating characteristic curve (AUC-ROC), sensitivity and specificity. There were 68,889 patients in the NSAIDs cohort with mean age 76 years and 54% were female. 1882 patients were admitted for acute coronary syndrome and 5405 patients died within one year after their first supply of NSAIDs. The multi-layer neural network, gradient boosting machine and support vector machine were applied to build various classification models. The gradient boosting machine achieved the best performance with an average AUC-ROC of 0.72 predicting ACS and 0.84 predicting death. Machine learning models applied to linked administrative data can potentially improve adverse outcome risk prediction. Further investigation of additional data and approaches are required to improve the performance for adverse outcome risk prediction.

## Introduction

Non-steroidal anti-inflammatory drugs (NSAIDs) are extensively prescribed for pain relief^1^. A large number of structurally diverse NSAIDs with similar therapeutic effects have been developed and NSAIDs belong to the most widely used pharmacological drugs, both over the counter (OTC) and by prescription^2,3^. However, their potential association with cardiovascular (CV) adverse outcomes are also well known. Multiple previous studies have reported an increased risk of CV events from the use of NSAIDs^1,3–6^. For example, Rofecoxib, one of the NSAIDs we investigated, was withdrawn from the market in October 2004 after a randomised placebo-controlled trial showed an increased risk of CV events among users^5^. Importantly, the population commonly taking NSAIDs is elderly individuals who have a higher risk of adverse outcomes^1,3,7^.

Adverse outcomes in older patients are a major burden in society, resulting in severe morbidity, mortality and significant healthcare costs^8^. Older adults are nearly seven times more likely to be hospitalised due to drug-related problems than younger patients^8,9^. Thus, accurate risk prediction models for adverse outcomes of drugs are necessary in clinical practice to help doctors to reduce the risk in the elderly^10^. A large number of surveys aimed to identify the key factors increasing a person’s risk of adverse outcomes have been proposed^11,12^, but they are not suitable for predicting the individual risk of adverse events due to the considerable differences in diseases and drug history between patients. This motivates the machine learning based risk prediction model design based on patients’ comorbidity and medication history obtained from suitable data sources, preferably at the population level.

Machine learning is increasingly common in big data science, with rapid uptake for medical applications^13–16^. There are advantages in using machine learning in risk predictions based on a wide array of patient data^17,18^. These can be used as decision support tools to aid prescribing of drugs in clinical practice. On wider application, they can be used to predict the risk of adverse outcomes of drugs at the population level. The availability of population-based drug dispensing data from the Pharmaceutical Benefits Scheme (PBS) in Australia, when linked to hospital admissions and death, offers an ideal opportunity to identify adverse outcomes following medication use at the population level. Acute coronary syndrome (ACS), consisting of acute myocardial infarction and unstable angina, is one of the common adverse outcomes of NSAIDs^1,19^. Death is also important as studies have shown increased mortality associated with NSAIDs^20^.

The aim of this study was to build machine learning models to predict the risk of ACS and all-cause death in elderly patients who were dispensed NSAIDs in Western Australia. Our motivation was to apply this as a test case to determine the utility of machine learning at the population level using multiple linked administrative datasets. We included comorbidity history and medication history for model development. All records were from the PBS data linked with Hospital Morbidity Data Collection (HMDC) for hospital admissions, and death register dataset in Western Australia. We compared the performance of different machine learning models and analysed the impact of features on the machine learning model.

## Methods

**Figure 1:**
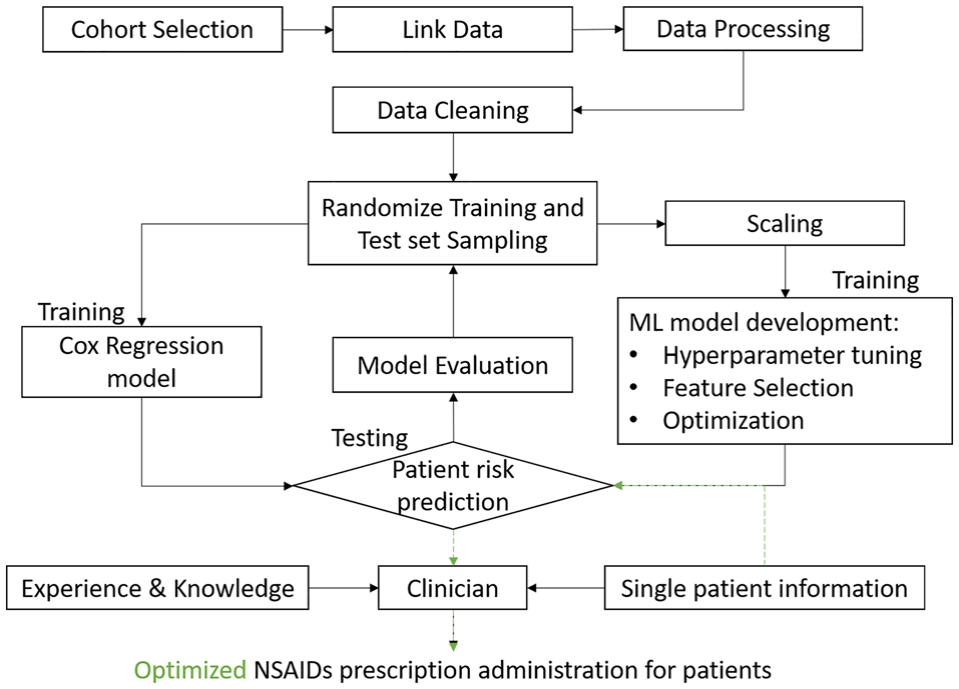
The machine learning workflow and contribution of our study. The figure was created using Microsoft PowerPoint 365, available from: https://office.microsoft.com/PowerPoint.

We used administrative data and built machine learning models to predict ACS and mortality risk of patients who had NSAIDs dispensed from pharmacies upon presenting a prescription. As shown in Fig. 1, we selected our cohort from the linked administrative data, and then processed and cleaned the data for our risk prediction models. We then randomly split the data into training and testing sets, built the machine learning models, evaluated their performance, and optimized the performance through hyperparameter tuning and feature selection.

### Data sources

The study datasets were a subset of population-level data consisting of public and private hospital admissions for heart disease in Western Australia during 2003–2008 from the HMDC, with linked admission records back to 1980 and forward to 2014^21^. These were linked to matching records from the Western Australian death registry to 2014, and PBS data from mid-2002 to mid-2011 from the Australian Department of Human Services. The HMDC and mortality data are 2 of the core datasets of the Western Australian Data Linkage System^22^. The PBS dataset contains patient-level information for medications dispensed from PBS-registered pharmacies in the community and in hospitals, including details such as drug name and strength, quantity supplied, and supply date.

### Inclusion criteria and selection

**Figure 2:**
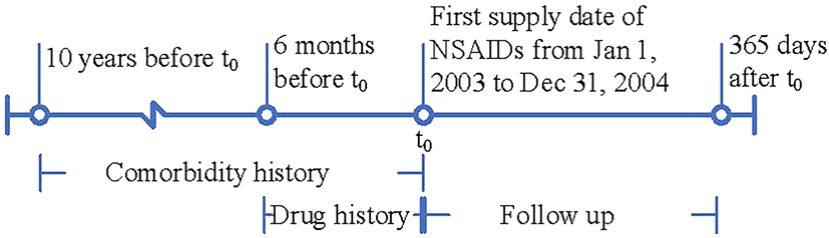
Timeline for study cohort showing history, exposure and follow-up periods. The first supply date for the COX-2 inhibitors or ibuprofen within years 2003 and 2004 was defined as ${{\varvec{t}}}_{0}$. The figure was created using Microsoft Visio 365, available from: https://products.office.com/en/visio/flowchart-software.

We identified patients supplied with NSAIDs at least once between 1 Jan 2003 and 31 Dec 2004 and aged 65 or above, from the PBS dataset. All the drugs were identified by their Anatomical Therapeutic Chemical (ATC) code. This period, corresponding to rofecoxib being withdrawn from the market in October 2004, ensured that we could capture all the records of NSAIDs. The PBS dataset records medications where the government pays a share of the drug cost, and does not include records where the patients pays for the drug in full. Previous research has shown that patients aged 65 or above are mostly concessional beneficiaries, and their dispensing records in the PBS data are mostly complete^23^. Furthermore, most of the patients taking NSAIDs are also elderly and adverse outcomes are more common and serious in the elderly. Thus, the age of the patients in the study was restricted to 65 and above. Figure 2 shows the study timeline. The study patients were those with dispensing records between 1 Jan 2003 and 31 Dec 2004. Comorbidity history was identified using a 10-year lookback period, and drug history was determined using a 6-month lookback. ACS and all-cause death were identified within one year after the first NSAID supply date.

### Input features

The features in our model consist of (1) patient demographic information, (2) comorbidity history, and (3) drug history. Demographic information includes age, gender, marital status, and Indigenous ethnicity. These are very common features in medical records and are considered to be strongly related to the patient’s health. Age was defined at the first supply date of the NSAIDs for the study cohort. Marital status and Indigenous ethnicity were defined at the last admission before the patients’ first NSAID supply. Comorbidity history and drug history are recorded based on the timeline design (Fig. 2). The history of comorbidities was determined from the diagnosis codes based on the International Classification of Diseases (both ICD-9-CM and ICD-10-AM) in the hospital admission dataset with a 10-year lookback period from the first supply date (see detailed list of ICD codes in Supplementary Table S1). Comorbidities included 13 features: ischaemic heart disease, hypertension, atrial fibrillation, diabetes, chronic obstructive pulmonary disease, peripheral vascular disease, stroke, chronic kidney disease, cancer, dementia, depression, heart failure, and cardiomyopathy. We included comorbidity history as continuous variables representing the frequency of previous admissions of each comorbidity within the 10-year lookback. Drug history was identified using a 6-month look back from the first supply date of the cohort using the PBS data, and drugs were grouped into 16 features corresponding to the first character of the ATC code. We also included the history of NSAIDs as 13 features corresponding to the 13 NSAIDs investigated. Drug history was presented as continuous variables representing the total number of medications supplied to patients.

### Outcomes

**Figure 3:**
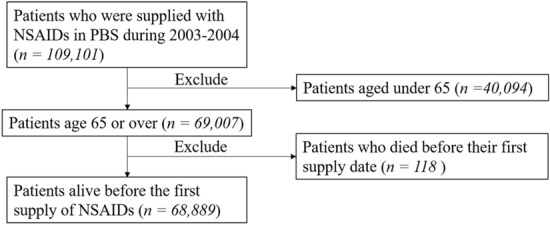
Flowchart showing identification of the study cohort. ACS, acute coronary syndrome. The figure was created using Microsoft PowerPoint 365, available from: https://office.microsoft.com/PowerPoint.

We focused on the patients’ risk of ACS and all-cause death in our study, as previous studies have presented the CV risks of NSAIDs^1,3–6,19,20^. ACS admission was identified from the principal discharge diagnosis field from the HMDC records using ICD-10-AM code I20.0 for unstable angina and I21 for myocardial infarction. We also classified patients who died due to coronary heart disease causes (ICD-10-AM I20-I25) as ACS. Patients who had drug supplies recorded after they died were excluded. (Fig. 3). Deaths were identified from the death registry. We also looked at a composite outcome, including both ACS admissions and all-cause death. Follow-up of patients began after their first supply date and finished at 365 days after the first supply date. In all the records we obtained, there were some patients with the same input features but different outcomes (with or without the event), which interfered with the prediction results. Therefore, we excluded these records before training the machine learning models.

### Machine learning method

We developed three machine learning models for risk prediction: gradient boosting machine (GBM), multi-layer neural network (MLNN) and support vector machine (SVM). These machine learning models perform well in clinical risk prediction^16,18,24,25^. However, there is no literature exploring their performance in risk prediction for NSAIDs in a population-level study. Further details of GBM, MLNN (Supplementary Fig. S1) and SVM are described in the Supplementary File. All analyses and model building were done with Python version 3.7 and relevant libraries, including scikit-learn^26^, and Keras^27^.

The predictive performance of models was compared by calculating sensitivity, specificity, and the area under the receiver operating characteristic curve (AUC-ROC). We used the Youden index^28^ to identify the optimised threshold for the ML model predictions that would achieve a balanced sensitivity and specificity. Other measures, such as positive predictive value (PPV), negative predictive value (NPV) and F1 score were not calculated. These depend on the prevalence of the outcomes being measured, which is low for the ACS and death outcomes associated with use of NSAIDs, and will lead to distorted values for PPV, NPV and F1 score. However, sensitivity and specificity are not affected by the prevalence of the outcomes being measured.

For all models, we randomly split the dataset using different random states and calculated their mean performance matrices and their 95% confidence intervals from training and evaluating the models 50 times. Once the outperforming model was identified, we conducted a sensitivity analysis using the individual NSAIDs testing set (excluding NSAIDs with less than *100* test samples) and measured its prediction performance. The randomization and repeated experiments also reduce the potential for confounding by generating groups that are fairly comparable with respect to the confounding factors^29,30^. The model was then compared with the Cox regression model based on the same features to validate our modelling and performance. We built two cox regression models, with one of them using the same continuous variables as we had in the machine learning models. The other Cox model was built on the same features, but all features were binary variables. Feature importance plots were generated by GBM for inspection.

### Ethics approval

Human Research Ethics Committee approval was obtained from the University of Western Australia (RA/4/1/8065), the WA Department of Health (2014/11), and the Australian Department of Health (XJ-16). We were granted a waiver of informed consent. All methods were carried out in accordance with relevant guidelines and regulations.

## Results

### Cohort characteristics

Figure 3 shows the results of each step in identifying the study cohort from the dataset. There were 109,101 patients supplied with NSAIDs during 2003 and 2004, and 40,212 were excluded due to age < 65 years or they died before the first supply (Fig. 3). Therefore, we identified 68,889 patients in the cohort with more than 40% as users of celecoxib and 35% users of rofecoxib. Table 1 shows patient characteristics for the study groups. The mean age was 76 years, and more than 50% of the cohort was female. More males developed ACS, and older patients were more likely to develop ACS or die within the follow-up period. History of cardiovascular diseases such as ischaemic heart disease and heart failure were more common among patients who developed ACS than those with no ACS. The frequency of comorbidity history was higher in patients who died during the follow-up.

**Table 1:** Characteristics of the study cohorts for NSAIDs during 2003–2004.

Features	Total cohort	ACS		All-cause death	
	No. of patients (%) n = 68 889	No. of patients (%) n = 2757 (4.0%)	p-value	No. of patients (%) n = 5405 (7.8%)	p-value
Age (years, mean[SD])	76.0 (7.2)	78.8 (8.0)	< 0.0001	80.9 (7.8)	< 0.0001
Female	37 389 (54.3)	1324 (48.0)	< 0.0001	2733 (54.3)	< 0.0001
Celecoxib	29 774 (43.2)	1204 (43.7)	0.6	2373 (43.9)	0.3
Rofecoxib	24 432 (35.5)	953 (34.6)	0.3	1851 (34.3)	0.051
Indometacin	4088 (5.9)	180 (6.5)	0.2	370 (6.9)	0.004
Sulindac	286 (0.4)	11 (0.4)	0.9	29 (0.5)	0.1
Diclofenac	11 660 (16.9)	422 (15.3)	0.02	705 (13.0)	< 0.0001
Diclofenac, combinations	18 (0.03)	< 5	< 0.0001	< 5	0.2
Piroxicam	3414 (5.0)	130 (4.7)	0.6	203 (3.8)	< 0.0001
Meloxicam	12 982 (18.8)	434 (15.7)	< 0.0001	686 (12.7)	< 0.0001
Ibuprofen	4305 (6.3)	161 (5.8)	0.4	319 (5.9)	0.3
Naproxen	5883 (8.5)	214 (7.8)	0.1	435 (8.1)	0.2
Ketoprofen	1951 (2.8)	65 (2.4)	0.1	122 (2.3)	0.008
Tiaprofenic acid	398 (0.6)	10 (0.4)	0.1	34 (0.6)	0.6
Fenamates	46 (0.07)	< 5	0.9	< 5	0.4
**Comorbidity history**					
Ischemic heart disease	14 445 (21.0)	1031 (37.4)	< 0.0001	1150 (21.3)	0.6
Hypertension	1091 (1.6)	52 (1.9)	0.2	113 (2.1)	0.002
Atrial fibrillation	3468 (5.0)	198 (7.2)	< 0.0001	390 (7.2)	< 0.0001
Diabetes	3779 (5.5)	186 (6.8)	0.003	409 (7.6)	< 0.0001
COPD	3659 (5.3)	255 (9.3)	< 0.0001	624 (11.5)	< 0.0001
PVD	3226 (4.7)	277 (10.1)	< 0.0001	474 (8.8)	< 0.0001
Stroke	2545 (3.7)	164 (6.0)	< 0.0001	425 (7.9)	< 0.0001
Chronic kidney disease	1337 (1.9)	78 (2.8)	< 0.0001	159 (2.9)	< 0.0001
Cancer	3049 (4.4)	98 (3.6)	0.02	747 (13.8)	< 0.0001
Dementia	386 (0.6)	27 (1.0)	0.003	140 (2.6)	< 0.0001
Depression	831 (1.2)	43 (1.6)	0.08	94 (1.7)	0.0002
Heart failure	2474 (3.6)	335 (12.2)	< 0.0001	629 (11.6)	< 0.0001
Cardiomyopathy	136 (0.2)	9 (0.3)	0.1	19 (0.4)	0.008
**Drug history**					
Drug group A	42 495 (61.7)	1868 (67.8)	< 0.0001	4032 (74.6)	< 0.0001
Drug group B	22 848 (33.2)	1214 (44.0)	< 0.0001	2352 (43.5)	< 0.0001
Drug group C	57 953 (84.1)	2389 (86.7)	0.0002	4496 (83.2)	0.05
Drug group D	13 710 (19.9)	546 (19.8)	0.9	1128 (20.9)	0.06
Drug group G	6924 (10.1)	243 (8.8)	0.03	536 (9.9)	0.7
Drug group H	12 275 (17.8)	520 (19.6)	0.01	1494 (27.6)	< 0.0001
Drug group J	30 857 (44.8)	1336 (48.5)	< 0.0001	3151 (58.3)	< 0.0001
Drug group L	3192 (4.6)	131 (4.8)	0.8	585 (10.8)	< 0.0001
Drug group M	29 698 (43.1)	1152 (41.8)	0.2	1935 (35.8)	< 0.0001
Drug group N	46 410 (67.4)	2075 (75.3)	< 0.0001	4517 (83.6)	< 0.0001
Drug group P	5829 (8.5)	290 (10.5)	< 0.0001	593 (11.0)	< 0.0001
Drug group R	13 747 (20.0)	640 (23.2)	< 0.0001	1456 (26.9)	< 0.0001
Drug group S	21 652 (31.4)	933 (33.8)	0.005	1919 (35.5)	< 0.0001
Drug group V	2554 (3.7)	109 (4.0)	0.5	261 (4.8)	< 0.0001

*SD* standard deviation, *COPD* chronic obstructive pulmonary disease, *PVD* peripheral vascular disease, *ACS* acute coronary syndrome. Drug groups: *A* alimentary tract and metabolism, *B* blood and blood forming organs, *C* cardiovascular system, *D* dermatologicals, *G* genito urinary system and sex hormones, *H* systemic hormonal preparations, *J* anti-infectives for systemic use, *L* antineoplastic and immunomodulating agents, *M* musculo-skeletal system, *N* nervous system, *P* antiparasitic products, *R* respiratory system, *S* sensory organs, *V* various.

### Performance of machine learning models

Table 2 shows the performance of different ML models as averages of the model sensitivity, specificity and AUC-ROC from training and evaluating the models 50 times. Among the algorithms examined, we found that GBM using features including age, sex, marital status, Indigenous ethnicity, comorbidity history and drug history as continuous variables achieved the best performance in predicting the risk of ACS (AUC 0.72, 95% CI 0.71–0.73). It slightly outperformed MLNN (AUC 0.71, 95% CI 0.70–0.71) and SVM (AUC 0.710, 95% CI 0.707, 0.712). The GBM had an average sensitivity of 61% (95% CI 60–63%) and an average specificity of 72% (95% CI 70–73%) using cutoffs selected by the Youden index. Machine learning models achieved similar performance in predicting all-cause mortality (AUC 0.84) and composite outcome (AUC 0.78) using the same features. We also compared machine learning models with a Cox regression model based on the same features. The Cox regression model had a lower average AUC (0.659 95% CI 0.656–0.662).

**Table 2:** Performance of machine learning models and Cox regression measured by sensitivity, specificity, and AUC-ROC.

Models	Performance metrics	ACS (95% CI)	All-cause death (95% CI)	ACS or All-cause death (95% CI)
GBM	Sensitivity	0.61 (0.60, 0.63)	0.78 (0.78, 0.79)	0.68 (0.67, 0.69)
	Specificity	0.72 (0.70, 0.73)	0.74 (0.73, 0.75)	0.75 (0.74, 0.75)
	AUC-ROC	0.72 (0.71, 0.72)	0.837 (0.836, 0.839)	0.780 (0.778, 0.781)
MLNN	Sensitivity	0.61 (0.60, 0.63)	0.76 (0.75, 0.76)	0.69 (0.68, 0.70)
	Specificity	0.70 (0.69, 0.71)	0.76 (0.75, 0.77)	0.75 (0.74, 0.75)
	AUC-ROC	0.70 (0.70, 0.71)	0.834 (0.833, 0.836)	0.778 (0.776, 0.780)
SVM	Sensitivity	0.61 (0.60, 0.62)	0.74 (0.73, 0.75)	0.70 (0.69, 0.71)
	Specificity	0.72 (0.71, 0.73)	0.75 (0.74, 0.75)	0.73 (0.72, 0.74)
	AUC-ROC	0.710 (0.707, 0.712)	0.813 (0.812, 0.814)	0.777 (0.776, 0.779)
Cox Regression	Sensitivity	0.62 (0.60, 0.64)	0.71 (0.70, 0.72)	0.66 (0.66, 0.67)
	Specificity	0.63 (0.61, 0.65)	0.69 (0.67, 0.70)	0.66 (0.65, 0.66)
	AUC-ROC	0.659 (0.656, 0.662)	0.76 (0.75, 0.76)	0.711 (0.710, 713)
Cox regression*	Sensitivity	0.638 (0.602, 0.674)	0.726 (0.710, 0.742)	0.653 (0.641, 0.665)
	Specificity	0.66 (0.625, 0.694)	0.729 (0.712, 0.746)	0.728 (0.718, 0.739)
	AUC-ROC	0.695 (0.688, 0.702)	0.795 (0.793, 0.797)	0.750 (0.745, 754)

*Cox model with binary variables.

Table 3 shows the performance of GBM on predicting the outcomes in patients supplied with different NSAIDs. It achieved the highest AUC for patients supplied with sulindac while predicting their risk of ACS (AUC 0.84). Its performance in predicting the risk of ACS was lower for patients supplied with piroxicam (AUC 0.66). We found similar average AUC between different NSAIDs on all-cause mortality risk prediction, with a slightly lower AUC (0.79) for patients supplied with ketoprofen. The AUC was higher while predicting the risk of the composite outcome for patients supplied with sulindac and tiaprofenic acid.

**Table 3:** Risk prediction performance of GBM models (AUC-ROC 95% CI) for different NSAIDs.

NSAID	ACS	All-cause death	ACS or All-cause death
Indometacin	0.71 (0.70, 0.72)	0.81 (0.80, 0.81)	0.77 (0.77, 0.78)
Sulindac	0.84 (0.78, 0.89)	0.82 (0.80, 0.84)	0.82 (0.80, 0.84)
Diclofenac	0.67 (0.66, 0.68)	0.80 (0.79, 0.80)	0.74 (0.74, 0.75)
Piroxicam	0.66 (0.65, 0.68)	0.80 (0.79, 0.81)	0.73 (0.72, 0.74)
Meloxicam	0.70 (0.69, 0.70)	0.80 (0.80, 0.81)	0.75 (0.74, 0.75)
Ibuprofen	0.71 (0.70, 0.73)	0.82 (0.81, 0.82)	0.76 (0.75, 0.77)
Naproxen	0.71 (0.70, 0.72)	0.82 (0.81, 0.82)	0.77 (0.77, 0.78)
Ketoprofen	0.71 (0.69, 0.73)	0.79 (0.78, 0.80)	0.73 (0.72, 0.74)
Tiaprofenic acid	0.77 (0.72, 0.82)	0.85 (0.83, 0.87)	0.83 (0.80, 0.85)
Rofecoxib	0.710 (0.705, 0.714)	0.821 (0.819, 0.823)	0.78 (0.77, 0.78)
Celecoxib	0.72 (0.71, 0.72)	0.811 (0.809, 0.813)	0.772 (0.770, 0.774)

*NSAID* non-steroidal anti-inflammatory drug, *GBM* gradient boosting machine.

### Feature importance

**Figure 4:**
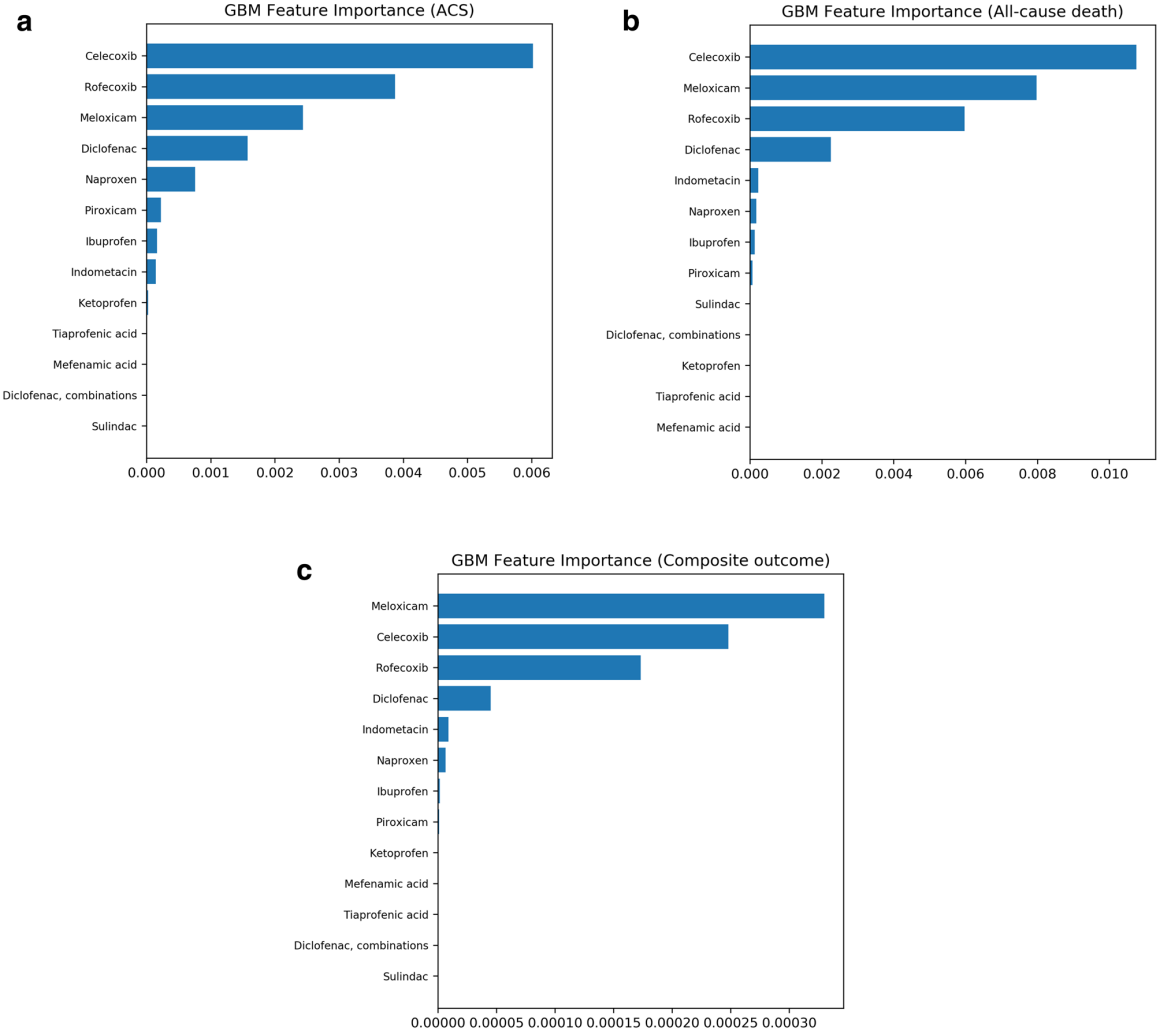
Ranking of NSAID feature importance from the GBM prediction models for adverse cardiovascular outcomes controlling for age, sex, comorbidity history and drug history. (**a**) Feature importance for ACS; (**b**) Feature importance for all-cause death; (**c**) Feature importance for the composite outcome (ACS or all-cause death). The figure was created using scikit-learn^26^.

Figure 4 shows the ranked feature importance for predicting adverse CV outcomes by GBM controlling for age, sex, comorbidity history and drug history. After controlling for these confounders, cyclooxygenase-2 (COX-2) inhibitors (rofecoxib, celecoxib and meloxicam) were ranked highest among all NSAIDs for predicting the risk of ACS and death (Fig. 4a,b). Naproxen, ibuprofen and ketoprofen were ranked lower compared with COX-2 inhibitors. Due to the small sample size of some NSAIDs such as tiaprofenic acid and mefenamic acid, their relative feature importance was at the bottom of the list. Similar results were found for the composite outcome (Fig. 4c). As shown in Supplementary Fig. S2A–C, confounding features were prominent, with age the most important predictor among all the features. History of cardiovascular diseases such as ischaemic heart disease and heart failure were also ranked high for predicting ACS, followed by drug group Cardiovascular system (C) and Nervous system (N). Cancer and heart failure history were important features associated with death, as well as drug group (N), and Musculo-skeletal system (M).

## Discussion

This study presents a set of machine learning models for predicting the risk of ACS and all-cause death after dispensing of NSAIDs using data from PBS, HMDC and death in Western Australia. We focused specifically on elderly patients (age ≥ 65 years) who had at least one NSAID supply. The prediction is based on the features including age, sex, medication history and disease history, which are routinely collected in administrative data. This approach encompasses a wide array of patients to reflect the population of patients taking NSAIDs in Western Australia. The machine learning based predictive models showed greater sensitivity, specificity and AUC-ROC values compared with the classical Cox-regression approach. GBM presented the best predictive performance for the machine learning models we tested.

Several studies have reported the risk of adverse outcomes with NSAIDs, and rofecoxib was withdrawn from the market due to its increased risk of CV outcomes. Our models predict ACS, all-cause death and composite outcome. The performance for predicting death was the best with AUC-ROC values ranging from 0.76 (Cox regression) to 0.84 (GBM). This demonstrates that the predictive models built based on administrative data work well and can predict the risk of death. The performance of the ACS risk prediction was lower, with AUC ranging from 0.66 (Cox) to 0.72 (GBM). The performance may be limited by the low event rate of ACS (4%), which makes the class distribution highly imbalanced. As shown in Table 2, GBM has slightly outperformed MLNN and SVM for predicting the risks of ACS, and SVM for predicting the risks of ACS and death. This difference may result from the nature of the boosting power in GBM, which is an ensemble method using many trees to make a decision as it gains power by repeating itself. MLNN is also a powerful model as it can learn complex data representations from underlying data, but is prone to overfitting^31^. Other studies have also found GBM can result in higher prediction accuracies compared with MLNN and SVM^32,33^. We considered the range of AUC-ROC we measured to be of moderate to high accuracy in predicting the risk of ACS or death in this population. While an ideal precision would be an AUC-ROC > 0.90, such high values are not easy to achieve in medical applications of machine learning due to the variations in patient characteristics we see in humans. Furthermore, this is our initial investigation on the potential for machine learning models to be applied for prediction of ACS and all-cause death using population-level administrative data. Further work needs to be done to determine if model performance can be improved, especially if other datasets can be added at the population level. We acknowledge that the outputs from the machine learning models do not necessarily suggest a causal link between the drug and the ACS admission or death. Instead, its purpose is to create an alert so that humans (clinicians, researchers, administrators) can investigate further and make a decision on whether the risk requires clinical or regulatory action. Hence, the machine learning application here will have clinical value as a decision support tool.

Risk prediction models have been used on different data sources (e.g. electronic medical record, administrative data) to identify risk of adverse outcomes for drugs. For example, predicting opioid overdose risk on administrative data with opioid prescriptions using deep neural networks and GBM^34^, predict adverse drug reactions from ICD-10 codes using machine learning models^35^ and comparing logistic regression with machine learning in predicting the risk of death from drug intoxication^36^. The AUC-ROC of the models from these studies ranged from 0.69 to 0.91. Our study made use of multiple linked administrative datasets, focusing on drugs and outcomes, and our machine learning risk prediction models achieved a range of AUC-ROC from 0.70 to 0.84. This is consistent with the performance attained in the previous studies reported above. Moreover, these studies found that the machine learning approach did not show better performance than a classical generalised regression approach^17,37^. However, our machine learning models performed better than the Cox regression models. This could be because most of the input features in our model were continuous variables, and machine learning models outperform on complex variables.

To our knowledge, there are no studies that explore the predictive capabilities of machine learning models for ACS and all-cause death in patients supplied with NSAIDs. Our study has several strengths. The risk prediction model we developed can be used to identify specific CV adverse outcomes of NSAIDs. The models can inform doctors on which NSAID has the lowest risk of these CV outcomes based on individual patient’s medication history and disease history. Moreover, our models have been developed using population-based datasets to identify patients with a high risk of adverse outcomes.

Our study found that the inclusion of demographic features such as marital status, Indigenous ethnicity from linked hospital admissions data improved the performance of the prediction models. The average AUC was similar for predicting ACS (AUC 0.71). However, the performance was higher while predicting the risk of all-cause mortality (AUC 0.81 vs 0.84) and composite outcome (AUC 0.77 vs 0.78), with no overlap in their confidence intervals. Previous studies have shown that marital status is associated with adverse cardiovascular outcomes and mortality was higher in an unmarried population^38,39^. Studies have also shown that Indigenous Australians have a greater risk of cardiovascular disease and death^40,41^.

We extracted additional features from the hospital admissions dataset, including patients’ previous length of stay (days) in the hospital for each comorbidity, and the number of days patients spent in intensive care units (ICU) before their first supply. This set of features were presented as continuous variables. We included this set of features to test whether it would improve the risk prediction. However, there were no performance gains by adding continuous variables such as length of hospital stay of previous comorbidities and days in ICU. The AUCs of all the outcomes were similar to models that did not include these extra features. Hence, we dropped these features to reduce model complexity.

In our study, we observed minimal performance improvement when using binary variables for comorbidities or drug history, indicating the presence of comorbidities and history of drugs. However, ML models achieved better performance than Cox regression when we used continuous variables for total counts of medication history and comorbidity history. This may be because machine learning approaches do not assume linearity for a predictor-outcome association. They are more adept at generating predictions based on continuous variables^42^.

Our machine learning model ranked COX-2 inhibitors higher among other NSAIDs for ACS risk prediction. Multiple previous studies have reported an increased risk of CV events from the use of selective COX-2 inhibitors^1,3–6^. Rofecoxib was withdrawn from markets based on evidence that showed an increased risk of ACS^5^. Naproxen and ibuprofen have been reported in several studies to be NSAIDs with less risk^1,43^. Compared with other popular NSAIDs, the rank of naproxen and ibuprofen was lower in our study, which is consistent with previous research. A previous study has confirmed that heart failure substantially increases the risk of death^44^. This verifies that our machine learning model is reliable in ranking feature importance as it showed the same relationship.

Despite the value of this study, there are some limitations. As with all administrative database studies, this study relies on the accuracy of administrative coding of diagnoses and procedures. However, the point of our study is that is makes use of multiple administrative datasets, which are large datasets that capture information at the population level. Despite whatever issues there may be with potential coding errors, we need these types of datasets to be able to adequately build a machine learning solution with potential for patient risk management. The PBS dataset did not include all dispensing supplies of NSAIDs such as ibuprofen, as this is also available over the counter. Moreover, the PBS dataset did not contain information about the actual drug dosage. Hence, in our study, we calculated the total number of supplied scripts rather than the dose used. In our study, we used state-level linked data to predict patients’ adverse CV outcomes after their NSAIDs supply. The models can be further extended to national linked data in the future. Also, for general applicability, the models can be potentially extended to other drugs or drug groups and different outcomes, and this can also be tested in future studies.

Implementing ML models on linked administrative data, including pharmacy claims (e.g. PBS), morbidity, and mortality has the potential to identify patients supplied with NSAIDs that may have a high risk of adverse CV outcomes. These can then be monitored closely by humans. Further investigation of additional data is required to validate the ML prediction performance on patients’ risk of CV adverse outcomes using population-level linked data. At this early stage our models were built with specific inputs from the research team, including looking at a specific follow-up period from NSAID use. However, further research will move towards more autonomy where the machine learning models will decide which drugs are potential problems and flag them for further investigation.

## Supplementary Information

Supplementary Information.
