# Supplementary material for: Machine learning risk prediction model for acute coronary syndrome and death from use of non-steroidal anti-inflammatory drugs in administrative data
Source: Sci Rep. 2021 Sep 15;11:18314. doi: 10.1038/s41598-021-97643-3 (PMC8443580; doi:10.1038/s41598-021-97643-3)
Supplement: Supplementary file 1 — Supplementary Information. [file 41598_2021_97643_MOESM1_ESM.pdf]

Machine Learning Risk Prediction Model for Acute Coronary Syndrome and Death from Use of Non-steroidal Anti-inflammatory Drugs in Administrative Data

Juan Lu[1,2,3,4], Ling Wang[2,5], Mohammed Bennamoun[3], Isaac Ward[2], Senjian An[6], Ferdous Sohel[3,7], Benjamin JW Chow[8], Girish Dwivedi[1,4,9,+], and Frank M Sanfilippo[2,*,+]

[1]Medical School, The University of Western Australia, Perth, 6009, Australia
[2]School of Population and Global Health, The University of Western Australia, Perth, 6009, Australia
[3]Department of Computer Science and Software Engineering, The University of Western Australia, Perth, 6009, Australia
[4]Harry Perkins Institute of Medical Research, Murdoch, 6150, Australia
[5]The State Key Laboratory of Integrated Service Networks, Xidian University, Xi'an, 710071, China
[6]School of Electrical Engineering, Computing and Mathematical Sciences, Curtin University, Bentley, 6102, Australia
[7]Discipline of information Technology, Murdoch University, Perth, 6150, Australia
[8]Division of Cardiology, University of Ottawa Faculty of Medicine and University of Ottawa Heart Institute, Ottawa, ON K1N 6N5, Canada
[9]Cardiology Department, Fiona Stanley Hospital, Murdoch, 6150, Australia

[*] corresponding author: frank.sanfilippo@uwa.edu.au
[+]G Dwivedi and FM Sanfilippo are co-senior authors

Multi-Layer Neural Network (MLNN) Architecture

All input features were scaled using the standard normalization method before fitting in the model, and all weights were initialized to small uniformly random values between 0 and 0.05. For each unit in the hidden layers such as g_j, we computed g_j based on units f_k from the previous layer: $g_j = \sigma(\sum_k u_{jk} f_k)$. $\sigma(x)$ function is the activation function, which is a rectifier function in the input and hidden layers, whilst a sigmoid function was applied to the output layer. As shown in Figure 3 (main paper), u_{jk} is the weight connected to unit g_j. Then we calculated the prediction $\hat{y}_i$ and calculated its binary cross-entropy loss. The adaptive moment estimation (Adam)[1] method was applied to minimize the loss by updating the weights connected to each unit. The batch size was 1400. Training continued to achieve minimal validation loss. To achieve the best performance on the test dataset, we examined the model learning curve. The MLNN model achieved its best training performance after 15 epochs. Finally, the model was tested on the test dataset. The output $\hat{y}_i$ was the predicted results of the test data, which are a column of floating point numbers between 0 and 1. The threshold was chosen based on the model performance, if $\hat{y}_i >$ threshold, it will be 1; otherwise, it will be 0.

Figure 1 shows a schematic of our fully connected MLNN. It consists of five layers: the input layer, three hidden layers, and the output layer. The three hidden layers contained 2250, 825 and 18 nodes respectively. The study dataset was divided into training (70%), validation (10%) and test sets (20%).

Our study dataset has a significant class imbalance issue because more than 90% of the patients were in the negative class (Class 0 or no outcome) thereby overwhelming the ability of the algorithms to predict the outcome class. To address the class imbalance in our data we applied balanced the class weights while training the model. All the sampling was accomplished using the imblearn library[2] in Python.

Gradient Boosting

The goal of a gradient boosting classifier is to create a sequence of weak learners (i.e. decision trees) to minimize the loss function. It was trained on 75% of the total cohort and tested on the remainder. We implemented a grid search and constrained 450 weak learners with learning rate 0.01, max depth 10, minimum number of samples equals 700, minimum number of samples at leaf node 50, subsample 0.8, and max feature set as ‘sqrt’. The model was implemented by the scikit-learn package in Python.

Support Vector Machine

Support vector machine is a popular binary classification technique. It aims to build a hyperplane between two classes, with the maximal distance between the hyperplane and the nearest points from two classes. The dataset was split into training (75%) and testing set (25%). Through grid search, we set C to 3 with radial basis function (RBF) kernel and used ‘scale’ gamma. We applied ‘balanced’ class weight as the data is highly imbalanced.

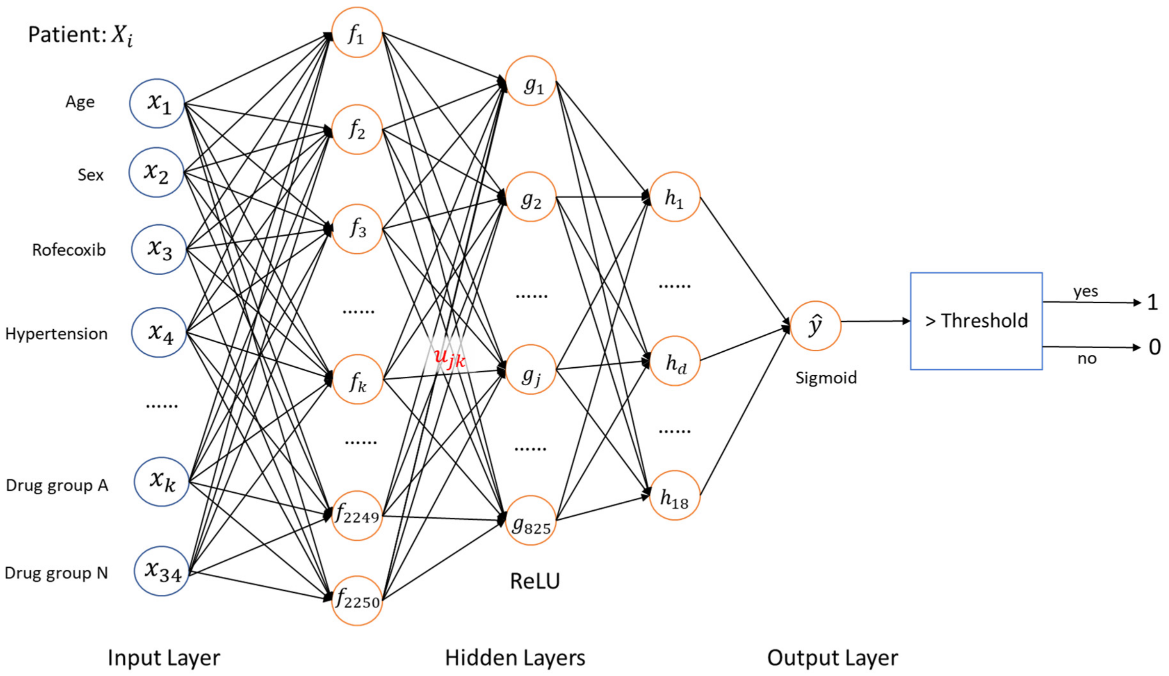

Figure 1: Overview of our multilayer neural network, demonstrating prediction for patient $\boldsymbol{x_i}$. For each unit in each hidden layer such as $\boldsymbol{g_j}$, compute $\boldsymbol{g_j}$ based on units $\boldsymbol{f_k}$ from the previous layer. Then predict $\hat{\boldsymbol{y}}_i$ and calculate the binary cross-entropy loss. To minimize the loss of the model, an Adaptive Moment Estimation (Adam) optimizer was applied to update the connected weights $\boldsymbol{u_{jk}}$ for each unit in each hidden layer such as $\boldsymbol{g_j}$. The training process continues to achieve minimal validation loss. The figure was created using Microsoft PowerPoint 365, available from: https://office.microsoft.com/PowerPoint

ReLU $=\max(0, x)$; Sigmoid $= \frac{1}{1+e^{-x}}$

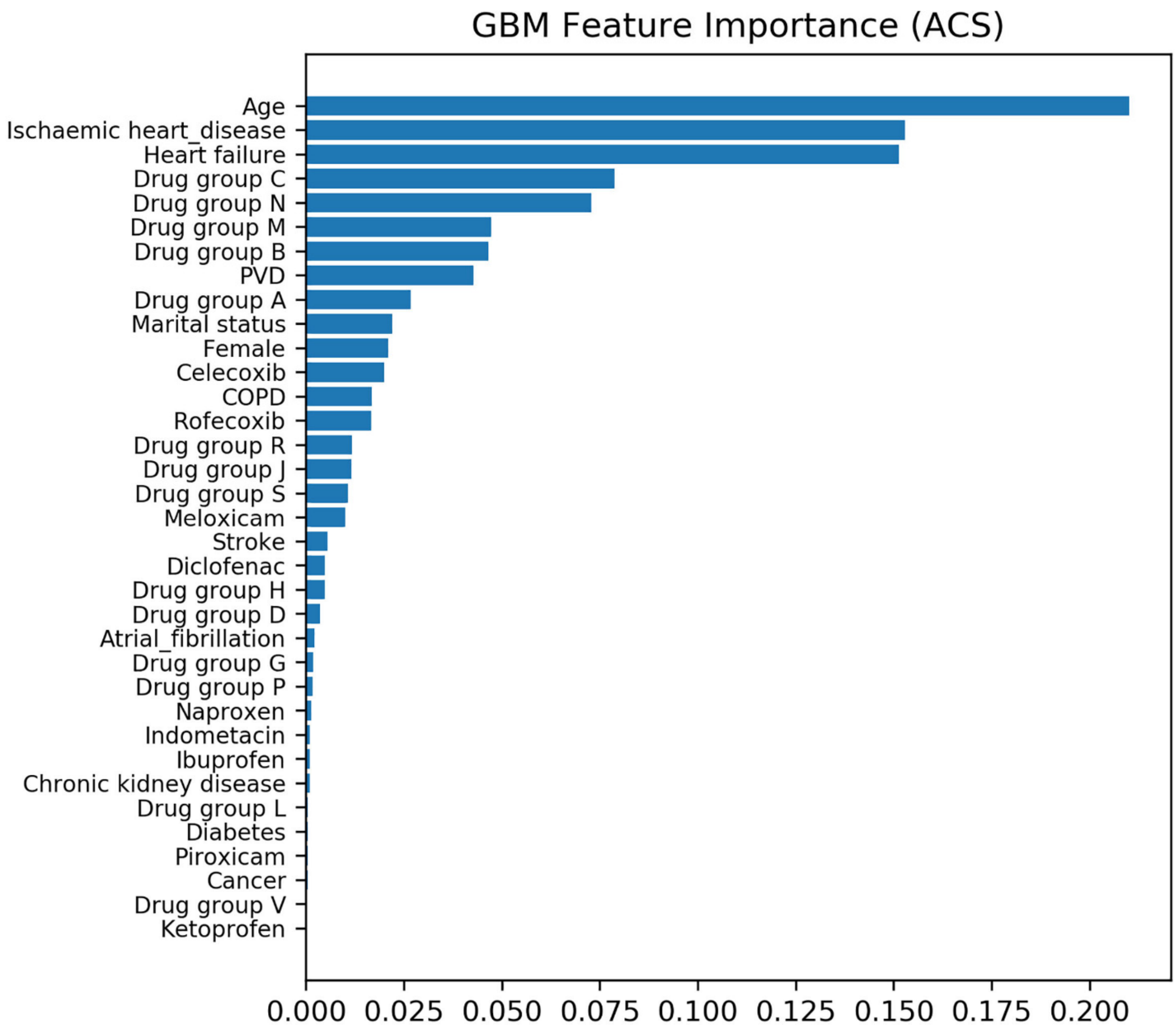

Figure 2A: Feature importance of the prediction model for adverse outcomes: for ACS.

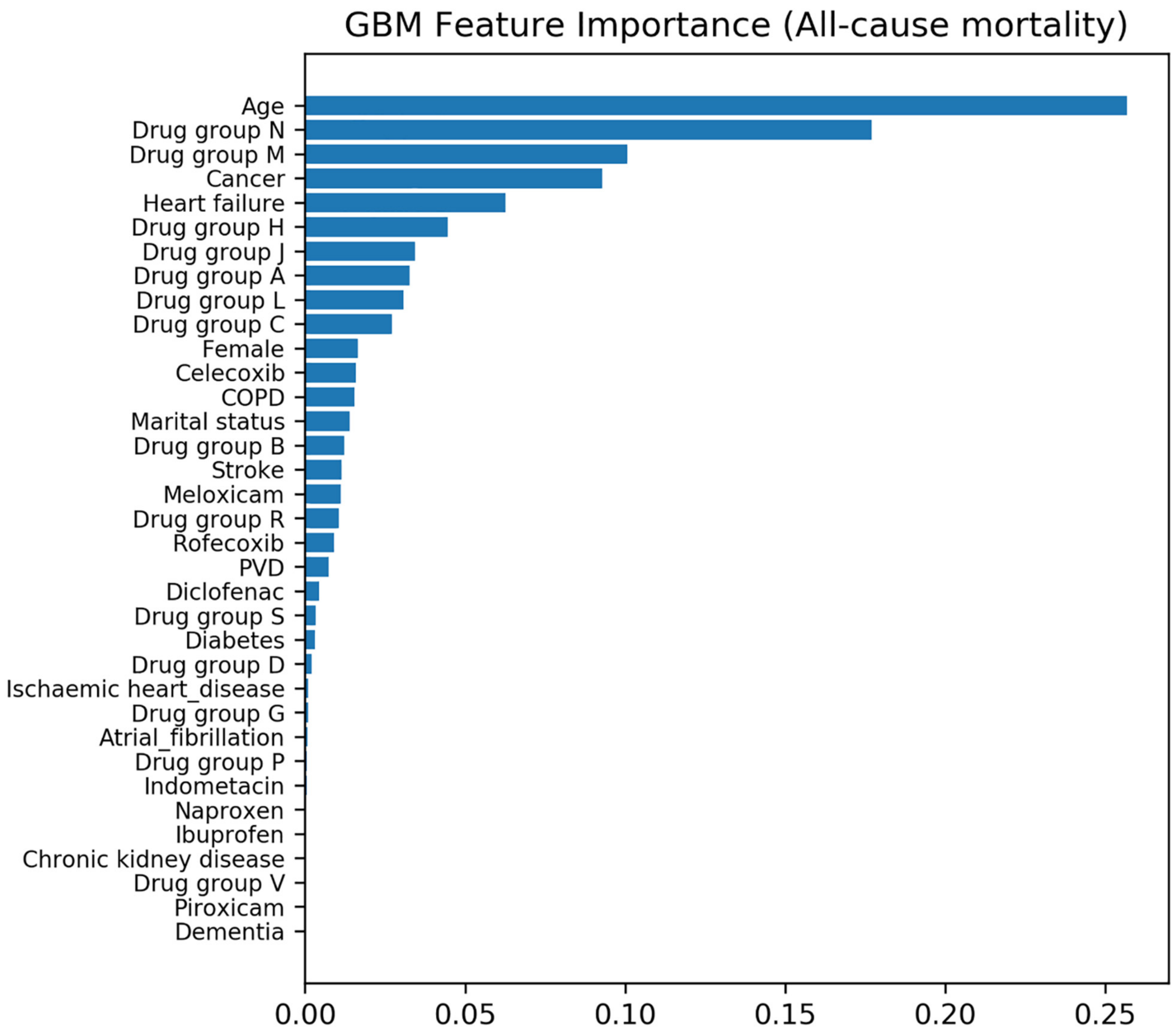

Figure 2B: Feature importance of the prediction model for adverse outcomes: for all-cause death.

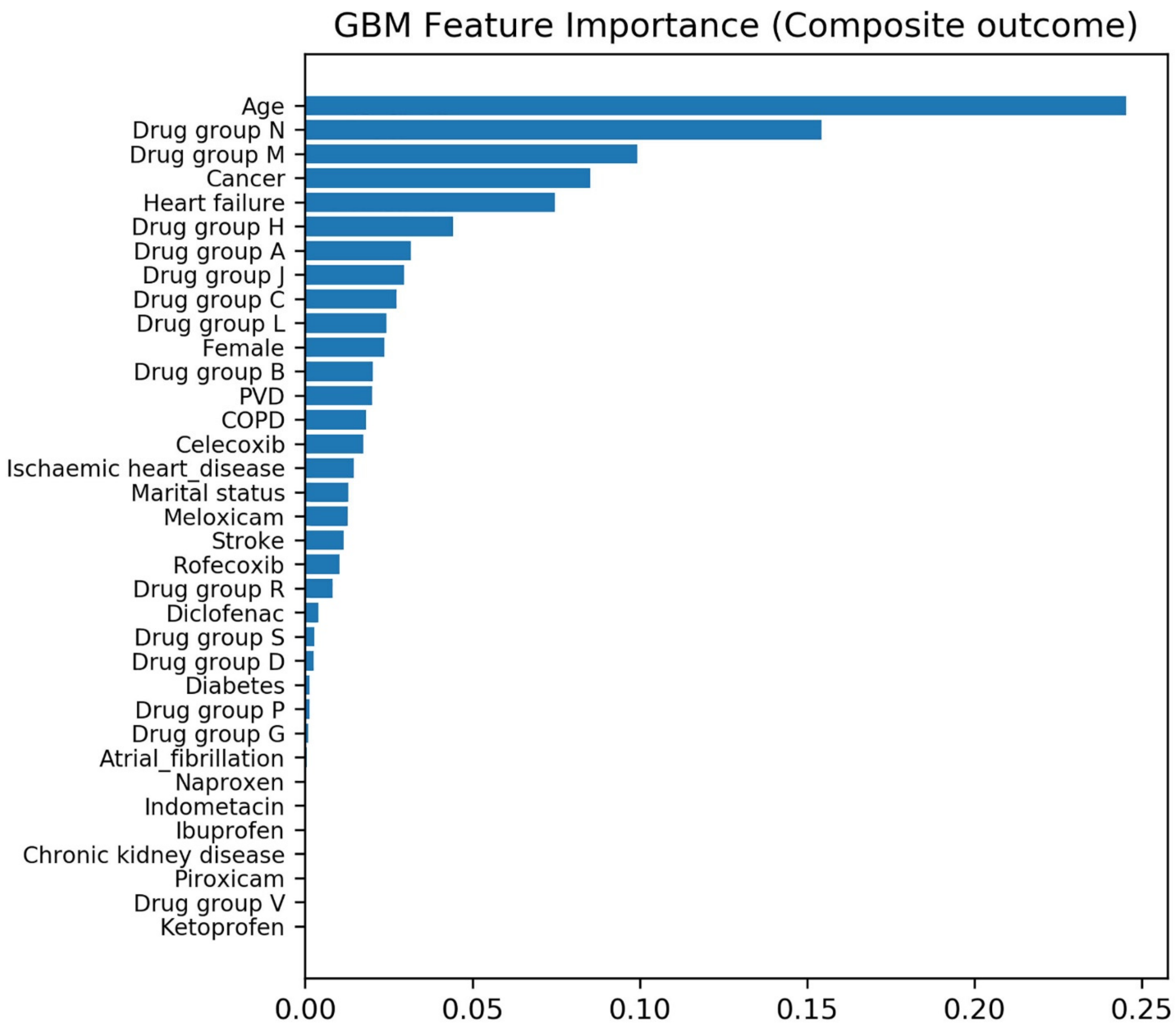

Figure 2C: Feature importance of the prediction model for adverse outcomes: for composite outcome (ACS or all-cause death).

Table S1: ICD codes for comorbidities.
ICD-9-CM, International Classification of Diseases, Ninth Revision, Clinical Modification; ICD-10-AM, International Classification of Diseases and Related Health Problems, Tenth Revision, Australian Modification.

Comorbidity	ICD-9-CM	ICD-10-AM
Ischaemic heart disease	410-414	I20-I25
Hypertension	401-405	I10-I15
Atrial Fibrillation	427.31	I48
Diabetes	250	E10-E14
COPD	490-496	J40-J46
PVD	440-442, 443.1, 443.9, 444, 447.1	I70-I72, I73.1, I73.9, I74, I77.1
Stroke	430, 431, 433, 436	I60, I61, I63, I64
Chronic Kidney Disease	250.3, 250.4, 250.42, 250.43, 580.0, 405.01, 405.11, 405.91, 405.02, 405.12, 405.92, 580.89, 580.9, 580.4, 582.4, 583.4, 599.7, 581.9, 582.0, 582.1, 582.2, 582.4, 582.89, 583.0, 583.1, 583.2, 583.6, 583.7, 583.89, 583.9, 580.81, 581.89, 582.81, 583.81, 587, 590.00, 590.01, 590.80, 590.9, 590.2, 590.81, 586, 588.0, 588.1, 588.8, 588.9, 589.0, 589.1, 589.9, 590.3, 593.0, 593.1, 593.2, 593.81, 593.82, 593.89, 593.9, 593.6, 753.0, 753.10, 753.11, 753.12, 753.13, 753.14, 753.15, 753.16, 753.17, 753.19, 753.2, 753.4, 753.3, 996.73, 996.81, 585.1, 585.9, 403, 404, I12, I13, 404.12, 404.13, 404.92, 404.93	E10.2, E14.2, I15.0, I15.1, N00, N01, N02, N03, N04, N05, N06, N07, N08, N11, N12, N14, N15, N16, N18, N19, N25, N26, N27, N28, N39.1, N39.2, Q60, Q61, Q62, Q63, T82.4, T86.1, Z49.0, Z49.1, Z49.2, Z94.0, Z99.2
Cancer	15, 16, 140, 141, 142, 143, 144, 145, 146, 147, 148, 149, 170, 171, 172, 196, 197, 198, 199, 200, 201, 202, 203, 204, 205, 206, 207, 208	C0, C1, C4, C5, C6, C7, C9, C20, C21, C22, C23, C24, C25, C26, C30, C31, C32, C33, C34, C37, C38, C39, C40, C41, C45, C46, C47, C48, C49, C50, C51, C52, C53, C54, C55, C56, C57, C58, C80, C81, C82, C83, C84, C85, C86, C87, C88
Dementia	290	F00, F01, F02, F03
Depression	311	F31, F32, F33, F34, F38
Heart Failure	428	I50
Cardiomyopathy	425	I42, I43

References

1. Kingma, D. P. & Ba, J. Adam: A Method for Stochastic Optimization. *ArXiv14126980 Cs* (2017).
2. Kotsiantis, S., Kanellopoulos, D. & Pintelas, P. Handling imbalanced datasets: A review. 12 (2006).
